# Use of the Left Ventricular Internal Dimension at End-Diastole and the E-Point Septal Separation Ratio in the Prediction of the Left Ventricular Systolic Function in Patients with Midrange and Reduced Ejection Fractions: A Pilot Study

**Published:** 2019-10

**Authors:** Kahraman Cosansu, Harun Kilic, Ayca Turer Cabbar, Erdinc Hatipsoylu, Bilgehan Karadag, Ramazan Akdemir

**Affiliations:** 1 *Department of Cardiology, Education and Research Hospital, Sakarya University, Adapazarı, Turkey.*; 2 *Department of Cardiology, Yeditepe University, Istanbul, Turkey.*; 3 *Department of Cardiology, Afsin State Hospital,* *Kahramanmaras, Turkey.*; 4 *Department of Cardiology, Cerrahpasa Medical Faculty, Istanbul University, Istanbul, Turkey.*

**Keywords:** *Heart Ventricles*, *Ventricular function, left*, *Heart failure*

## Abstract

**Background: **The aim of this study was to investigate the ability of a new index, namely the left ventricular internal dimension at end-diastole/mitral valve E-point septal separation (LVIDd/EPSS), to predict the left ventricular (LV) systolic function and to compare its performance with that of the EPSS index and to investigate the correlation between the LVIDd/EPSS and the left ventricular ejection fraction (LVEF).

**Methods: **The current study recruited 142 patients who presented to the Cardiology Clinic of Sakarya University Education and Research Hospital and were followed for heart failure (HF).M-mode measurements of the EPSS and the LVIDd were recorded in the parasternal long-axis view.

**Results: **Totally, 142 HF patients with midrange ejection fraction (HFmrEF) and reduced ejection fraction (HFrEF) were enrolled in the study. There was a significantly correlation both between the EF and the EPSS and between the EF and the LVIDd/EPSS (P<0.001). In both HFmrEF and HFrEF groups, the correlation between the LVIDd/EPSS and the EF was more significant than was the correlation between the EPSS and the EF (P<0.001). The results of the linear regression analysis indicated that the LVIDd/EPSS was an independent predictor of the HFmrEF and the HFrEF (P<0.001). In the patients with EPSS≤12, there was a significant association between the EF and the LVIDd/EPSS (P<0.001) but not between the EF and the EPSS(P>0.05). The receiver operating characteristic curve analysis showed that the LVIDd/EPSS predicted advanced HF with 87% sensitivity and 72% specificity, using a cutoff value of 3.35,and it predicted the HFrEF (EF<40%) with 84% sensitivity and 81% specificity, using a cutoff value of 3.75.

**Conclusion: **The LVIDd/EPSS may allow certain clinicians, especially beginners and emergency department physicians, to assess the LVEF when other methods are not available or questionable.

## Introduction

Heart failure (HF) comprises a wide range of patients, from those with a normal left ventricular ejection fraction (LVEF) (typically considered ≥50%) and HF with a preserved LVEF (HFpEF) to those with a reduced LVEF (typically considered <40%) (HFrEF). Transthoracic echocardiography is the method of choice for the assessment of the LV myocardial systolic function. The best approach to evaluate the cardiac function in the emergency department setting has not been determined. Measures for the calculation of the LVEF are difficult to perform and time-consuming, which limits their use in the emergency department.^1^ Most emergency department physicians are not trained in the quantitative calculation of the LVEF. A limited number of studies in the literature suggest that visual prediction by emergency department physicians correlates with the quantitative and semiquantitative methods of estimating the cardiac function.^2^ The major limitations of visual prediction for evaluating the LVEF are observer dependency and subjectivity.^3^

An alternate method for estimating the LVEF is the mitral valve E-point septal separation (EPSS). Similar to the left ventricular internal dimension at end-diastole (LVIDd), the EPSS is a simple linear M-mode measurement obtained from the parasternal long-axis view. The amount of separation between the valve leaflet and the septum in early diastole is defined as the EPSS.^4^ The EPSS can generate a rapid quantitative measure of the LV function, especially when the acquisition of multiple breath-hold short-axis images proves difficult.^5^ Previous studies have demonstrated a high negative correlation between the EPSS and the LVEF, with the EPSS measurements of >7 mm indicating a poor LV function.^6^

The dimensions of the LV increase in both systole and diastole in patients suffering from HF. Many physiological factors such as body size and heart rate affect echocardiographic data. Due to the strong relationship between heart size and body size, cardiac dimensions differ among individuals. Nonetheless, misinterpretations of cardiac images, especially in the presence of segmental wall motion impairment and inadequate image capacity, can lead to inaccurate estimations of the LVEF. A previous investigation revealed a significant correlation between the LV end-diastolic dimensions and the body surface area in patients with HF.^7^ The same research also demonstrated that the basal LVIDd affected the EPSS, which was useful in predicting systolic dysfunction. 

The left ventricular mass (LVM), a well-established measure used to independently predict adverse cardiovascular events and premature deaths,^8^ is strongly associated with body size.^9^ The LV relative wall thickness (RWT) refers to the relationship between wall thickness and cavity size.^10^ The left ventricular mass index (LVMI), which is widely used to assess LV hypertrophy due to arterial hypertension, is an independent risk factor for ischemic strokes.^11^ In patients with HF, these parameters may change with differentiation in the LV geometry and structure. These parameters are calculated by various formulations. An easy parameter with a significant association with all of these, however, has yet to be found.

In this study, we aimed to investigate the ability of a new index, namely the LVIDd/EPSS, to predict the LV systolic function and to compare its performance with that of the EPSS using M-mode. Secondarily, we aimed to determine whether the LVIDd/EPSS measurements correlate with the calculated LVEF from comprehensive transthoracic echocardiography.

## Methods

This was an observational study of the association between the EPSS and the LVIDd/EPSS and the LVEF in HF patients undergoing comprehensive transthoracic echocardiography for any indication. The current study recruited 142 consecutive patients who presented to the Cardiology Outpatient Clinic of Sakarya University Education and Research Hospital, Sakarya, Turkey, and were followed up for midrange HF or advanced heart failure (AHF) between March 2017 and April 2017. An LVEF between 40% and 49% was considered heart failure with a midrange ejection fraction (HFmrEF), an LVEF<40% was classified as HFrEF, and an LVEF≤30% was defined as AHF. In addition to routine echocardiographic measurements, the EPSS was measured by M-mode in the parasternal long-axis view, if the LVEF was ≤50%. The present study also tested whether or not the LVIDd/EPSS is useful in predicting HFrEF and AHF. The EPSS and the LVIDd/EPSS were compared for the EF correlation in various groups. The relationships between the LVM, the LVMI, and the RWT and the LVIDd/EPSS were investigated in the study population. 

This study was approved by the local institutional ethics committee and conducted in accordance with the Declaration of Helsinki.

M-mode and 2D echocardiograms were recorded on a Vivid S5 ultrasound system (GE Medical System, Horten, Norway with a 3.5-MHz multifrequency transducer). To prevent systematic errors in obtaining or interpreting the echocardiograms, 2 noninvasive cardiologists obtained the echocardiograms. The EPSS was measured in millimeters (mm) as the minimum separation distance between the mitral valve anterior leaflet and interventricular septum in M-mode echocardiography. Two-dimensionally M-mode measurements of the LVIDd, as well as septal and posterior wall thickness, were recorded. The EPSS and the LVIDd measurements were performed by 2 different cardiologists, and the mean values were taken by at least 2 measurements for reducing interobserver and intraobserver variabilities. The modified Simpson rule was used for calculating the EF. The LVM was calculated using the formula recommended by the American Society of Echocardiography and was indexed to the body surface area, as follows: LVM=0.8 × 1.04([LVIDd + LVPWTd + IVSTd]^3^ - [LVDd]^3^) + 0.6, where IVSTd was the diastolic interventricular septal wall thickness and LVPWTd was the diastolic LV posterior wall thickness.^12^ The RWT was defined as the ratio of twice the posterior wall thickness and the LVIDd.

Patients who had atrial fibrillation, asymmetric septal hypertrophy, severe LV hypertrophy, severe valve diseases, mitral valve with rheumatismal involvement, and an LVEF≥50% were excluded from the study. 

All the statistical data were analyzed using SPSS 15.0 for Windows (SPSS Inc., Chicago, IL, USA). The continuous data were expressed as the mean±standard deviation, and the categorical data were expressed as percentages. The Kolmogorov–Smirnov test was performed to determine whether the continuous variables had a normal distribution. The χ^2^ or Fisher exact test was carried out for between-group comparisons of the qualitative variables, when appropriate. The independent *t*-test was applied for the normally distributed continuous variables, and the Mann–Whitney *U* test was used for the non-normally distributed continuous variables. Conventional correlation and regression analyses were employed to analyze the relationship between the LVEF and the LVIDd/EPSS. A receiver operating characteristic curve (ROC) was plotted in the reference population to analyze the predictive capacity of the LVIDd/EPSS and to determine optimal cutoff points for these indices. Probability values <0.05 were considered statistically significant in the analyses. 

## Results

In total, 142 patients (98 male and 44 female) with HFmrEF and HFrEF were enrolled in the study. The median age was 64.09±12.77 years. The LVEF ranged from 15% to 49% (33.31±9.90), the EPSS ranged from 8 to 28 mm (16.47±4.83), and the LVIDd ranged from 40 to 73 mm (55.76±7.71). The median LVIDd/EPSS was 3.54±0.65. The baseline demographic characteristics of the study population are depicted in Table 1.

**Table 1:** Baseline demographic characteristics of the study population*

	Study Patients with HF (N=142)
Age (y)	64.09±12.77
Gender (male)	98 (69.0)
EF (%)	33.31±9.90
Hypertension	51 (35.9)
Diabetes mellitus	24 (16.9)
Hyperlipidemia	29 (20.4)
Ischemic heart disease	52 (36.6)

HF, Heart failure; EF, Ejection fraction

* Data are presented as the mean±standard deviation or numbers (%).

**Figure 1:**
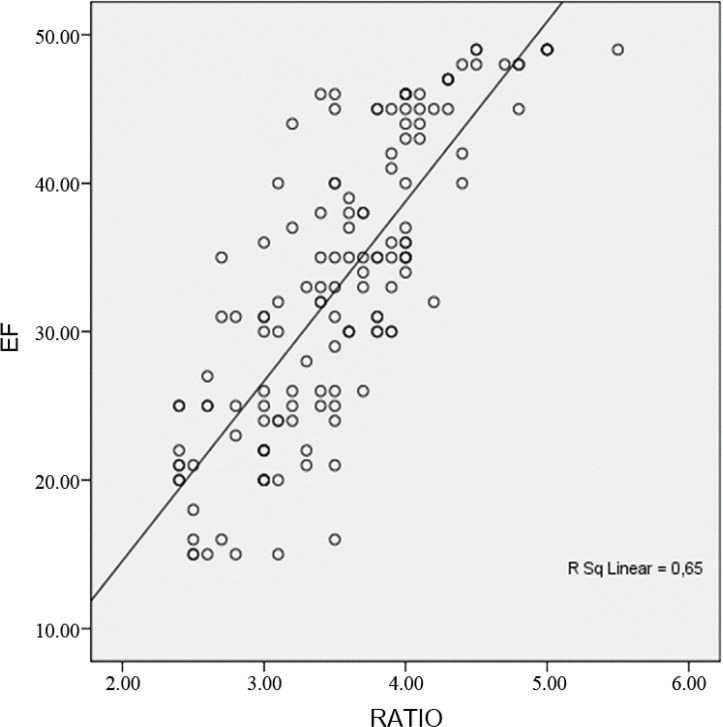
Figure depicts the scatterplot of the LVEF versus the ratio (LVIDd/EPSS) with a regression line. There was a highly significant correlation between the EF and the LVIDd/EPSS (*r*= .806) (P<0.001).

LVEF, Left ventricular ejection fraction; LVIDd/EPSS, Left ventricular internal dimension at end-diastole/mitral valve E-point septal separation

There was a strong significant correlation both between the EF and the EPSS (*r*= −0.773; P<0.001) and between the EF and the LVIDd/EPSS (*r*=0.806; P<0.001). In both the HFmrEF and HFrEF groups, the EF and the LVIDd/EPSS correlation was more significant than was the EF and the EPSS correlation (*r*=0.652 vs. *r*= −0.425 and *r*= 0.670 vs. *r*= −0.599; P<0.001). As was shown by the results of the linear regression analysis, the LVIDd/EPSS was significantly associated with the EF. The results of the linear regression analysis also indicated that the LVIDd/EPSS was an independent predictor of HFmrEF and HFrEF (β=0.806; P<0.001). Figure 1 shows the regression analysis between the EF and the LVIDd/EPSS (ratio).

The results of the analysis of the patients with an EPSS≤12 revealed a significant association between the EF and the LVIDd/EPSS (*r*=0.635; P<0.001) but not between the EF and the EPSS (P>0.05). In the patients with an EPSS>12, there was a significant correlation between the LVEF and the EPSS (*r*= −0.609) and the LVIDd/EPSS (*r*= 0.638; P<0.001).

In the nonischemic and ischemic HF subgroups, although there was a significant correlation between the EF and the EPSS (*r*=−0.707 and *r*=−0.791, respectively; P<0.001), the correlation between the EF and the LVIDd/EPSS was more significant (*r*=0.799 and *r*=0.801, respectively; P<0.001). 

When the groups were evaluated according to sex, there were no differences in the EF and the LVIDd/EPSS. However, the EPSS was significantly higher in the male HF patients (P*=*0.010). 

The results of the statistical analyses also revealed that the LVIDd/EPSS was statistically significantly correlated with the LVM, the LVMI, and the RWT (P<0.001 for all).

**Figure 2:**
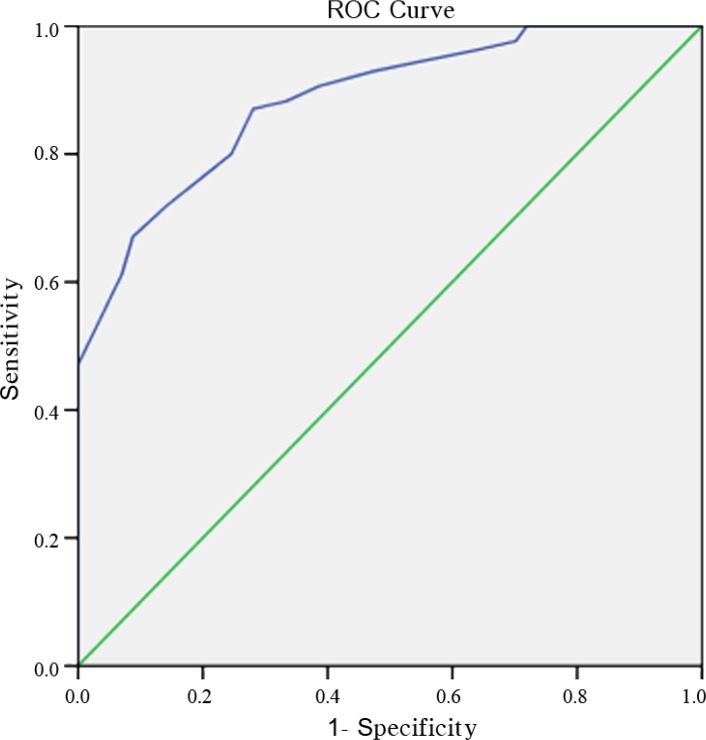
ROC analysis shows that the LVIDd/EPSS predicted AHF (EF≤30%), with 87% sensitivity and 72% specificity, using a cutoff value of 3.35 (ROC area=0.884, P<0.001, CI:0.83–0.94).

**Figure 3:**
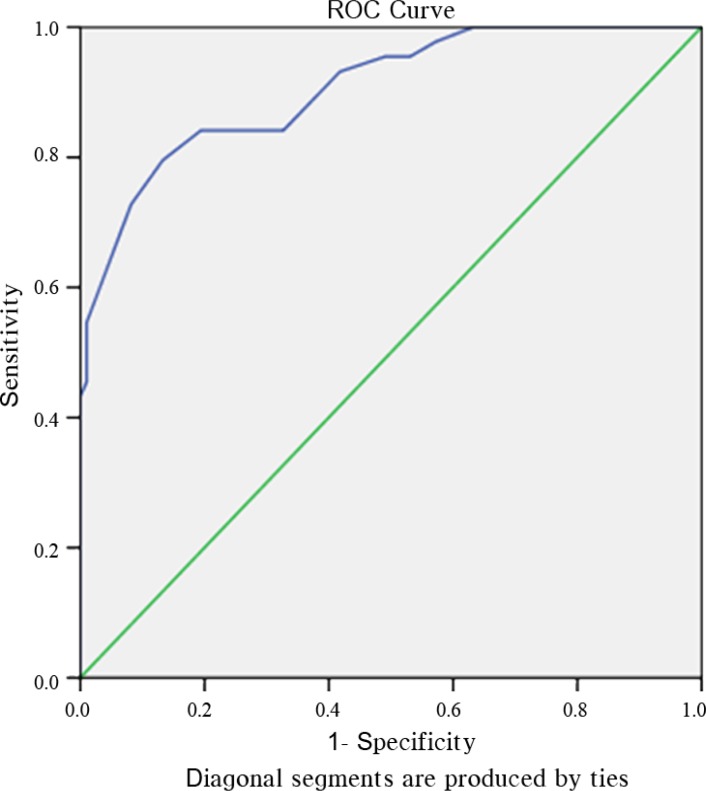
ROC analysis shows that the LVIDd/EPSS predicted HFrEF (EF<40%), with 84% sensitivity and 81% specificity, using a cutoff value of 3.75 (ROC area=0.905, P<0.001, CI: 0.85–0.96).

ROC, Receiver operating characteristic; LVIDd/EPSS, Left ventricular internal dimension at end-diastole/mitral valve E-point septal separation; HfrEF, Heart failure with a reduced ejection fraction

There was a significant difference between the HFmrEF (n=44) and HFrEF (n=98) groups in the EPSS and the LVIDd/EPSS (EPSS=11.9 mm vs. 18.4 mm; LVIDd/EPSS=4.1 vs. 3.2; P<0.001). The ROC analysis showed that the LVIDd/EPSS predicted AHF (EF≤30%), with 87% sensitivity and 72% specificity, using a cutoff value of 3.35 (ROC area=0.884, P<0.001, 95% CI: 0.83–0.94) (Figure 2). The LVIDd/EPSS predicted HFrEF (EF<40%), with 84% sensitivity and 81% specificity, using a cutoff value of 3.75 (ROC area=0.905, P<0.001, 95% CI: 0.85–0.96) (Figure 3).

## Discussion

The present study consisted of patients admitted to the cardiology outpatient clinic who had HFmrEF or HFrEF. The results revealed that although there was a correlation between the EF and the EPSS, there was a more significant correlation between the EF and the LVIDd/EPSS. The LVIDd/EPSS was an independent predictive marker of HFrEF. The LVIDd/EPSS was also correlated with the LVM, the LVMI, and the RWT, all of which are independent predictors of adverse cardiovascular events.

The visual estimation of the LVEF is commonplace and correlates well with ventriculography.^13^ The mitral valve EPSS is another easy-to-obtain echocardiographic parameter that correlates inversely with the LVEF. Secko et al.^14^ showed that junior emergency physicians could readily obtain the EPSS measurements that correlated with the visual estimates of the LVEF. By contrast, we used a quantitative calculation of the LVEF. Our results demonstrated a significant correlation between the EF and the EPSS in patients with HFmrEF and HFrEF. Moreover, contrary to prior literature, our results suggested that there was no correlation between the EF and the EPSS in patients with an EPSS≤12. 

In this study, we investigated whether the LVIDd/EPSS could serve as a substitute for the EPSS index and whether it could augment the sensitivity of this index. In all the study groups and subgroups, the LVIDd/EPSS showed a stronger correlation with the EF than with the EPSS. The group with an EPSS≤12, in which there was no correlation between the EPSS and the EF, the LVIDd/EPSS was strongly correlated with the EF. 

In dilated cardiomyopathy or other nonischemic diseases that cause AHF, ventricular dilatation develops and the chamber becomes rounder (spherical) as the disease progresses. Holler et al.,^15^ showed that the sphericity index, which is calculated by dividing the length of the LV through the width of the LV, does not appear to be more sensitive than the EPSS. Therefore, we did not use this index in our study.

This is the first study to suggest that the LVIDd/EPSS may predict the LVEF using linear regression. It is also the first study to examine the relationship between the EPSS and the LVIDd/EPSS and the EF in ischemic and nonischemic groups separately. In these groups, the LVIDd/EPSS was more significantly associated with the EF than was the EPSS. Furthermore, we examined the association between the EPSS and the LVIDd/EPSS and the EF in patients suffering from HF according to sex for the first time in this study and detected no significant difference. Nevertheless, the EPSS was larger in the males than in the females. This may be due to the greater body mass index and the LVIDd in men. Thus, the EPSS measurements in obese or thin individuals may lead to false EF estimates. The LVIDd/EPSS can remove the EF prediction errors that occur due to a nonstandard body mass index and the baseline LVIDd. In the present study, the ROC analysis showed that the LVIDd/EPSS predicted AHF (EF≤30%) and HFrEF (EF<40%), with high sensitivity and specificity.

The presence of a high LVM or a high LVMI is reportedly an independent predictor of increased cardiovascular morbidity and mortality, both in the general population and hypertensive populations.^16^ A previous investigation found independent associations between an increased RWT and the measures of systolic and diastolic LV functions, irrespective of the presence or absence of LV hypertrophy or hypertension.^17^ In the present study, we found a significant association between the LVIDd/EPSS and these parameters. These findings suggest that instead of evaluating the LVM, the LVMI, and the RWT separately, the LVIDd/EPSS measurement may provide sufficient information on all these parameters.

The retrospective design is the main limitation of the current study. In addition, this investigation was based on data from a single centre. Another weakness of this study is its relatively small sample size. In future studies, it may be more appropriate to include healthy individuals as well as a wide spectrum of patients suffering from HF (HFrEF, HFmrEF, and HFpEF). In addition, the associations between the LVIDd/EPSS and the LVEF in each category of the LV geometry (normal, concentric remodelling, concentric hypertrophy, and eccentric hypertrophy) merit further research.

## Conclusion

In the present study, the LVIDd/EPSS predicted systolic dysfunction as well as the EPSS. In addition to serving as an index of the LV function and dilatation, the LVIDd/EPSS was strongly correlated with the LVEF. Specifically, a ratio <3.75 predicted a poor LV function. The EPSS was more affected by the basal LVIDd. Therefore, a smaller basal LVIDd may result in a smaller EPSS increase. The LVIDd/EPSS accurately distinguished individuals with a normal LV function from those with an abnormal LV function, irrespective of the LV size. The LVIDd/EPSS, which can easily be calculated, appears to be quite useful for the prediction of systolic dysfunction, especially for beginners and emergency department physicians, when it is not possible to determine visually whether there is systolic dysfunction or not. The role of the LVIDd/EPSS in the prediction of systolic dysfunction requires further investigation in studies with a larger patient population.

## Notes:

***This paper should be cited as: ***Cosansu K, Kilic H, Turer Cabbar A, Hatipsoylu E, Karadag B, Akdemir R. Use of the Left Ventricular Internal Dimension at End-Diastole and the E-Point Septal Separation Ratio in the Prediction of the Left Ventricular Systolic Function in Patients with Midrange and Reduced Ejection Fractions: A Pilot Study. J Teh Univ Heart Ctr 2019;14(4):171-176.
